# Nanoscale thermal transport across an GaAs/AlGaAs heterostructure interface

**DOI:** 10.1063/1.5129629

**Published:** 2020-03-17

**Authors:** Matthew Gorfien, Hailong Wang, Long Chen, Hamidreza Rahmani, Junxiao Yu, Pengfei Zhu, Jie Chen, Xuan Wang, Jianhua Zhao, Jianming Cao

**Affiliations:** 1 Physics Department and National High Magnetic Field Laboratory, Florida State University, Tallahassee, Florida 32310, USA; 2 State Key Laboratory of Superlattices and Microstructures, Institute of Semiconductors, Chinese Academy of Sciences, P.O. Box 912, Beijing 100083, China; 3 Beijing Academy of Quantum Information Science, Beijing 100193, China; 4 School of Physical Science and Technology, Southwest Jiaotong University, Chengdu 610031, China; 5 Center for Ultrafast Science and Technology, Key Laboratory for Laser Plasmas (Ministry of Education) and School of Physics and Astronomy, Shanghai Jiao Tong University, Shanghai 200240, China; 6 Collaborative Innovation Center of Inertial Fusion Sciences and Applications (CICIFSA), Shanghai Jiao Tong University, Shanghai 200240, China; 7 Beijing National Laboratory for Condensed Matter Physics, Institute of Physics, Chinese Academy of Sciences, Beijing 100190, China; 8 College of Materials Science and Optoelectronics Engineering, University of Chinese Academy of Sciences, Beijing 100190, China

## Abstract

We studied the thermal transport across a GaAs/AlGaAs interface using time-resolved Reflection High Energy Electron Diffraction. The lattice temperature change of the GaAs nanofilm was directly monitored and numerically simulated using diffusive heat equations based on Fourier's Law. The extracted thermal boundary resistances (TBRs) were found to decrease with increasing lattice temperature imbalance across the interface. The TBRs were found to agree well with the Diffuse Mismatch Model in the diffusive transport region, but showed evidence of further decrease at temperatures higher than Debye temperature, opening up questions about the mechanisms governing heat transfer at interfaces between very similar semiconductor nanoscale materials under highly non-equilibrium conditions.

The drive to produce smaller, denser, and higher frequency electronics has culminated in the need for completely understanding and controlling thermal transport in devices at the nanometer scale.^1,2^ In nonmetallic systems, heat is conducted by phonons. As the characteristic length and timescales of devices become comparable to the phonon mean free path (MFP) and to the phonon relaxation time, the thermal transport can significantly depart from Fourier's Law due to the non-equilibrium characteristics, such as ballistic transport by heat carriers and concurrent energy exchanges among subsystems. In addition, as the device's dimension reduces, interfaces progressively dominate the phonon transport and can increase the overall thermal resistance by several orders of magnitude.^3–6^ Therefore, it is critical to understand the role of the interface in nanoscale thermal transport, particularly under these non-equilibrium conditions.

The heat flux *Q* across an interface of an area *A* for a temperature discontinuity ΔT is regulated by thermal boundary resistance^7^ (TBR) that is defined as $R_{B}=\Delta T\times A/Q$. Several recent studies show that *R_B_* not only depends on interface properties such as roughness and bond strength mismatch but also on the transport mechanisms in the thermal body.^1,8–10^ These studies attributed the non-Fourier phenomena in nanoscale thermal transport to dominant phonon-interface interactions and proposed that for a steady-state, heat equations based on Fourier's Law still hold if *R_B_* is adjusted according to the modified boundary conditions.^8,11,12^ In contrast, several other studies conclude that neither Fourier's Law nor the Cattaneo equation can apply at small scales and in fast transient processes, creating the necessity for ballistic-diffusive models or numerical methods based on the Boltzmann transport equation to be used.^13–15^ In order to benchmark these theoretical models and to improve our understanding of nanoscale heat transport, quantitative experiments with a relevant spatial and/or temporal resolution are of great importance.

Most of the previous experimental studies measuring TBR used the technique of Time-Domain Thermoreflectance (TDTR) or other similar optical methods.^16–18^ The validity of such a technique relies on the linear relation in the change in optical reflectivity and lattice temperature, which is only true for very small perturbations. Therefore, TDTR is suitable to study thermal transport under quasi-static conditions. Calorimeters^19^ and other techniques such as electrical resistance thermometry^20^ can measure heat or temperature very accurately, but do not have a temporal component, limiting them to experiments under steady-state conditions. On the other hand, ultrafast x-ray^21,22^ and electron diffraction techniques^23–26^ combine the atomic-level spatial and temporal resolution, which meet the requirements for nanoscale heat transport studies. Meanwhile, these techniques can directly monitor the lattice temperature, allowing them to explore the heat transport processes in highly non-equilibrium and non-steady conditions.

In this paper, we used ultrafast electron diffraction (UED) to study the thermal transport in a GaAs/AlGaAs heterostructure by directly monitoring the lattice temperature in the time-domain. The maximum lattice temperature change was 350** **K above room temperature. Using heat equations based on Fourier's Law with modified boundary conditions, *R_B_* was extracted and compared with the results of previous theoretical studies. We found that *R_B_* decreased with increasing temperature imbalance across the interface. Additionally, when heating the sample up to 650 K, our model fitting started to deviate from the data and the extracted *R_B_* could not be explained.

The experiment was conducted using our UED instrument^27^ arranged in standard Reflection High Energy Electron Diffraction (RHEED) geometry, as shown in Fig. 1(c). The laser pulses, with a pulse width of 40 fs and a wavelength of 800 nm running at 1 kHz, were split into pump and probe pulses. The pump beam, retaining 90% of the initial laser beam energy, was first sent through a motion stage and then focused onto the sample to initiate ultrafast heating. The induced transient lattice temperature was recorded by taking snapshots of RHEED patterns with probe electron pulses, which were generated by the frequency tripled probe optical pulses via the photoelectric effect. The time delay between the pump and probe beams was set by the linear stage. The diffraction patterns (DPs) were first intensified and then recorded using a cooled CCD camera. The experiments were performed under ultra-high vacuum (UHV) conditions with a base pressure of $4\times 10^{-10}$ Torr. Due to the geometrical and velocity mismatch^28,29^ between the pump and probe beams, the overall temporal resolution of the experiment was about 18 ps.

**FIG. 1.**
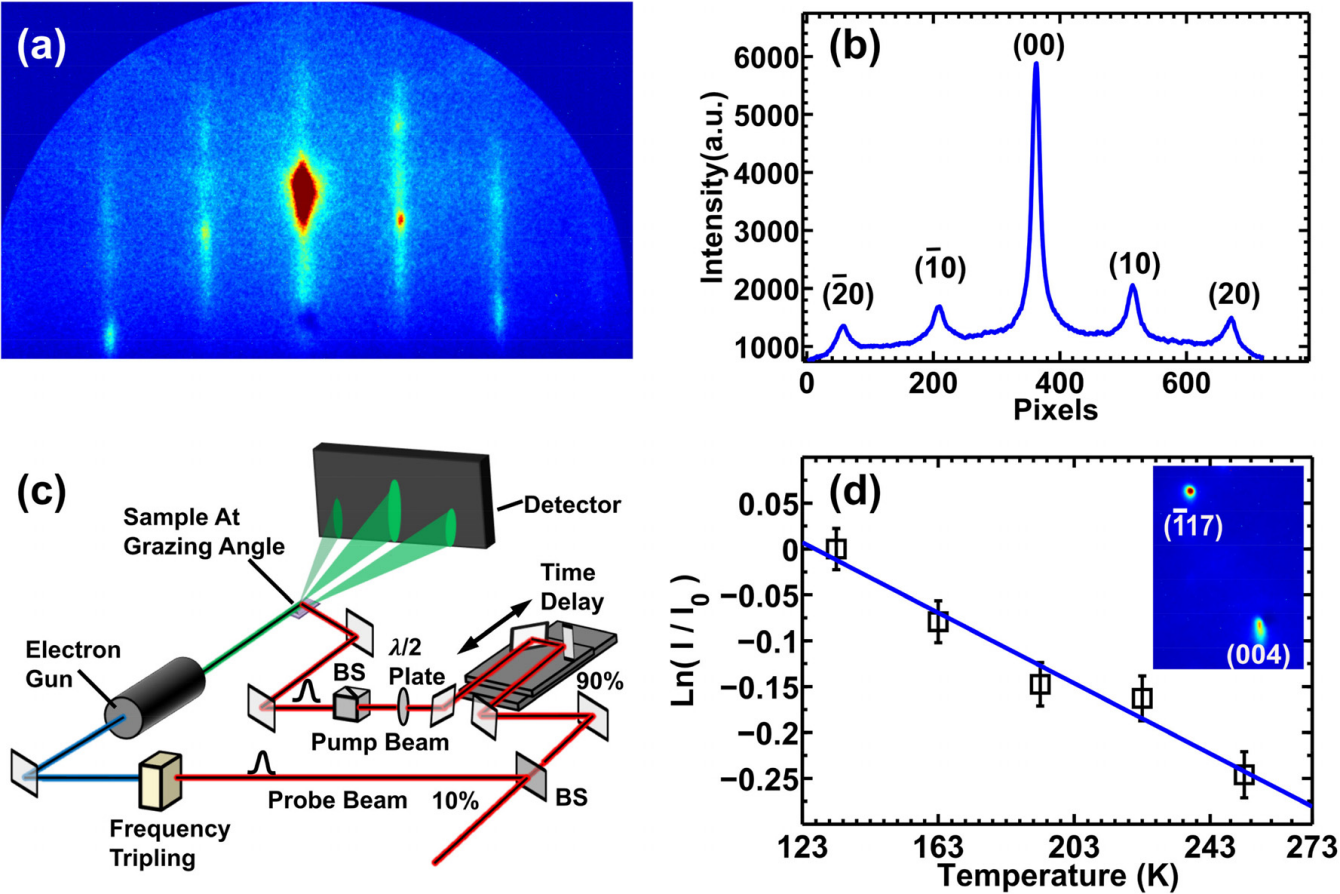
(a) DP of the GaAs nanofilm along the [110] zone axis and (b) the corresponding intensity profile taken along the horizontal axis. (c) Time resolved RHEED experimental setup: the pump pulse initiates excitation of the nanofilm sample under study and the subsequent surface dynamics are probed using a short probe electron pulse at a grazing angle. The time delay between the pump and probe pulses can be adjusted using an optical delay stage. (d) Results from a static measurement of the intensity change of the (−1, 1, 7) Bragg peak as a function of sample temperature. The blue solid curve is the linear fitting. The inset shows the (−1, 1, 7) and (0, 0, 4) diffraction spots.

The high quality GaAs/AlGaAs heterostructure, consisting of a 10 nm GaAs film on top of a 500 nm AlGaAs film, was grown using Molecular Beam Epitaxy on a thicker GaAs buffer with a (0, 0, 1) surface orientation. The bandgap of AlGaAs was carefully tuned to be 1.73 eV by giving a composition of Al_0.25_Ga_0.75_As, preventing the substrate from being excited by the 1.55 eV pump laser. This ensured that only the top 10 nm GaAs (1.42 eV bandgap) nanofilm was excited and heated by a large amount during the laser induced heating. Figure 1(a) shows a typical RHEED pattern of the top GaAs film after decapping the protective As layer at 400 °C and yielding an atomically clean GaAs surface. The intensity is integrated along each RHEED streak in the vertical direction, and the profile is taken along the horizontal axis as shown in Fig. 1(b). The diffuse scattering background is then subtracted, and each peak was fitted with either a Gaussian or Lorentzian line profile, yielding its intensity, peak center position, and width.

In order to calibrate the transient nanofilm temperature, a static measurement was first conducted by placing the sample on a heating-cooling stage and taking DPs as the temperature of the stage was varied. Figure 1(d) shows the results of the static experiments for the (−1, 1, 7) Bragg spot intensity change as a function of temperature. The (−1, 1, 7) peak was chosen because its higher momentum transfers (higher Miller indices) increase both the detection sensitivity and the probe penetration depth to roughly 1.2 nm. The diffraction peak intensity can be directly related to the sample temperature through the surface Debye–Waller factor (DWF) by

$$\frac{I}{I_{0}}\propto \text{exp}(-\frac{1}{3}\langle u^{2}(T)\rangle |K{|}^{2}),$$ (1)

where *I*_0_ is the intensity at a reference temperature, $\left\langle u^{2}(T)\right\rangle$ is the mean square displacement of an atom, which is linear to the lattice temperature change above 120 K,^30^ and ***K*** is the momentum transfer vector related to each diffraction peak.^31^

Following the static calibration, we conducted pump–probe experiments on the same sample at room temperature using four different pump fluences. The results are shown in Fig. 2, where the (−1, 1, 7) peak intensity change has already been converted to the temperature change using the static calibration curve. Considering that the optical penetration depth of 710 nm from the pump laser^32^ is much larger than the top film thickness, we can assume that the entire 10 nm GaAs nanofilm is instantaneously and uniformly excited. In addition, we can ignore the heat transport along the in-plane direction considering the much larger laser beam lateral dimension of 1 mm scale. Therefore, the thermal conduction can be modeled by a set of 1D heat equations along the film normal direction. The equations for the GaAs layer are

$$c_{1}\cdot \partial_{t}T_{1}(z,t)=\partial_{z}[\kappa_{1}\cdot \partial_{z}T_{1}(z,t)]+S(z,t),$$ (2)

$$\partial_{z}T_{1}(z,t){|}_{z=0}=0,$$ (3)

$$\kappa_{1}\cdot \partial_{z}T_{1}(z,t){|}_{z=d_{1}}=-\frac{T_{1}(d_{1},t)-T_{2}(d_{1},t)}{R_{B}},$$ (4)

$$T_{1}(z,0)=300\;K,$$ (5)

where *c*_1_ and *κ*_1_ are the volumetric heat capacity^33^ and the thermal conductivity of GaAs,^34^ and both depend on the film temperature $T_{1}(z,t)$. $T_{2}(z,t)$ is the temperature of the AlGaAs substrate, *d*_1_ is the 10 nm film thickness, and *S*(*z*, *t*) is the laser-induced heat source. The interface between the thicker GaAs (001) buffer and AlGaAs was not considered because the heat diffusion range within 100 ps is only several tens of nm. Even if the initial ballistic transport is considered, its range (100 nm) is still much smaller than the 500 nm thickness of the AlGaAs layer. The source term *S*(*z*, *t*) is set by the sample geometry, excitation condition, and subsequent relaxation dynamics of carriers and phonons. In general, the dynamics of carriers and phonons after optical excitation involves a few stages with different timescales. Initially, electrons are excited into conduction bands with about 0.13 eV excessive energy per electron–hole pairs. These hot carriers redistribute their energies among themselves through various carrier-carrier scattering processes. Particularly, via Auger scattering, a portion of the electrons and holes recombine non-radiatively, transferring their energy (1.42 eV) to the remaining ones. Meanwhile, the hot carriers relax their energy to the lattice, mainly through scattering with longitudinal optical (LO) phonons.^35–38^ However, since optical phonons have a very small sound velocity, significant heat transport would not occur until the energy further relaxed to acoustic phonons (phonon thermalization) in several ps.^37,39–44^ We simulated such a process by using a three-temperature-model^39,45,46^ (details not presented here) and found that within 10 ps, the carriers and lattice would reach the same temperature. Such a timescale was comparable to the temporal resolution of our experiment but much shorter than that of the observed thermal transport (several hundred ps). Therefore, *S*(*t*) can be treated as a Delta function in the time-domain, equivalent to setting an initial temperature distribution *T_init_* throughout the film. Thus, Eqs. (2)–(5) can be re-written as

$$c_{1}\cdot \partial_{t}T_{1}(z,t)=\partial_{z}[\kappa_{1}\cdot \partial_{z}T_{1}(z,t)],$$ (6)

$$\partial_{z}T_{1}(z,t){|}_{z=0}=0,$$ (7)

$$\kappa_{1}\cdot \partial_{z}T_{1}(z,t){|}_{z=d_{1}}=-\frac{T_{1}(d_{1},t)-T_{2}(d_{1},t)}{R_{B}},$$ (8)

$$T_{1}(z,0)=T_{\mathrm{init}}.$$ (9)

For the heat transport inside the AlGaAs layer, similar heat equations can be written as

$$c_{2}\partial_{t}T_{2}(z,t)=\partial_{z}[\kappa_{2}\partial_{z}T_{2}(z,t)],$$ (10)

$$\kappa_{2}\partial_{z}T_{2}(z,t){|}_{z=d_{1}}=-\frac{T_{1}(d_{1},t)-T_{2}(d_{1},t)}{R_{B}},$$ (11)

$$T_{2}(d_{1}+d_{2},t)=300\;K,$$ (12)

$$T_{2}(z,0)=300\;K,$$ (13)

where *c*_2_ and *κ*_2_ are the volumetric heat capacity^47^ and the thermal conductivity of the AlGaAs layer.^34,48^ *d*_2_ is the layer thickness of 500 nm. Equations (8) and (11) are the boundary conditions,^8^ which produce a temperature jump across the interface. Equations (6)–(13) are solved numerically using a forward time centered space (FTCS) finite difference method.^49^ To give an intuitive perception of the effect of TBR, the surface temperature change of a homogeneous sample of 510 nm with no interface (assuming only 10 nm on the surface was excited to match the initial condition of the real process) was first simulated by assuming $R_{B}=0$, equivalent to replacing Eqs. (8) and (11) with

$$\kappa_{1}\cdot \partial_{z}T_{1}(z,t){|}_{z=d_{1}}=\kappa_{2}\partial_{z}T_{2}(z,t){|}_{z=d_{1}},$$ (14)

and the results were plotted as the dotted curves shown in Fig. 2(a). As shown in Fig. 2(a), the timescale of the actual heat transport in sample is much slower than that of the simulation, indicating the significant role of the interface in impeding the heat flow. The values of *R_B_* under different pump fluences are extracted by fitting the solution of Eqs. (6)–(13) to the experimental data with *R_B_* as a floating parameter, and the fitting curves are shown in Fig. 2(a). For each fitting curve, *R_B_* is considered to be independent of temperature, which is elaborated later. The extracted values of *R_B_* are plotted in Fig. 2(b).

**FIG. 2.**
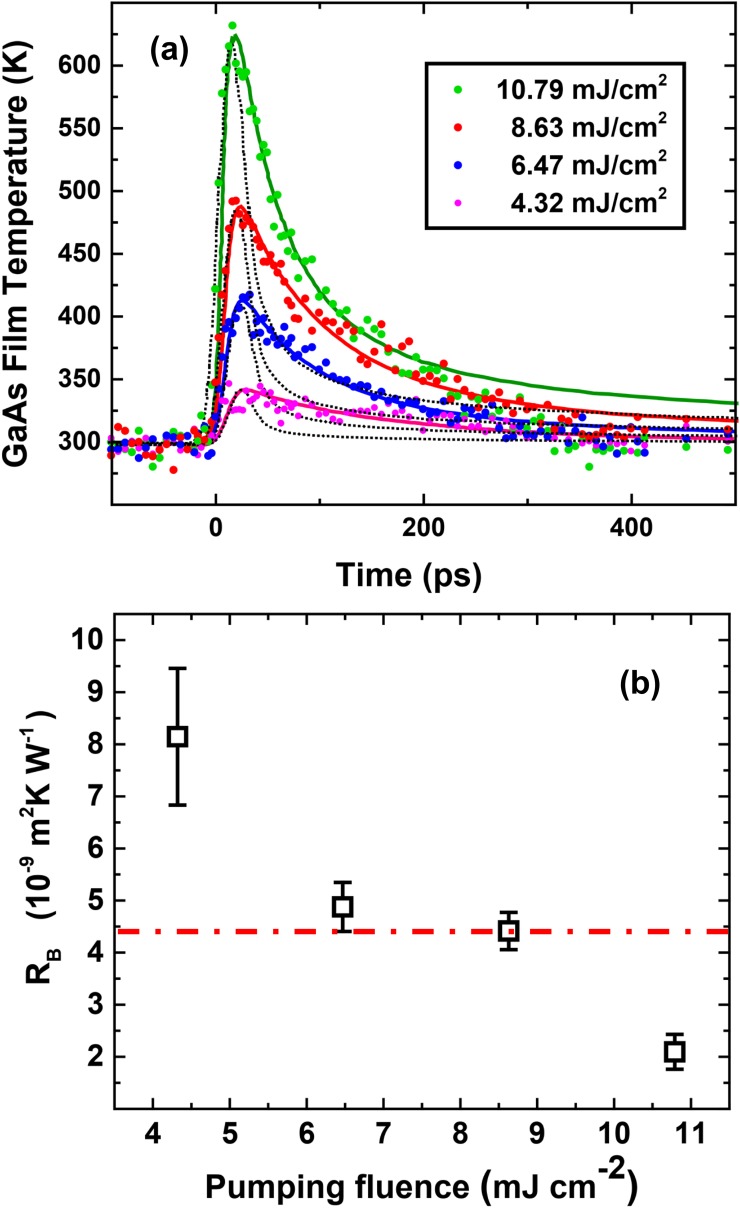
(a) Lattice temperature change as a function of time for four different pumping fluences. The dotted curves are numerical solutions of the diffusive heat transport equations assuming zero thermal boundary resistance, which are much faster than the experimental data. The solid curves are fitting with the thermal boundary resistance *R_B_* as a fitting parameter. (b) The extracted *R_B_*'s for the four pumping fluences. The values marked by the dotted-dash line are the calculated $R_{\mathrm{dif}}=4.4\times 10^{-9}$ m^2^ K W^−1^ for 300** **K, which decreased slightly to $4.3\times 10^{-9}$ m^2^ K W^−1^ at 600** **K.

It has been demonstrated in several studies that *R_B_* depends on both the property of the interface (roughness and bond-strength mismatch) and the transport mechanism (ballistic or diffusive) in the thermal conductor. More specifically, *R_B_* depends on the phonon transmission coefficient *α_ij_* (*ij* indicates the transmission direction from *i* material to *j* material), which in turn depends on the phonon branches, phonon frequencies, and incident angles.^1,50^ *α_ij_* is usually evaluated based on the acoustic mismatch model (AMM) or the diffuse mismatch model (DMM).^1^ In general, when the phonon wavelength is larger than the interface roughness, phonons can be treated as elastic waves that are either reflected or refracted at the interface as described by the AMM. In the other limit, phonons with wavelengths smaller than the interface roughness will be strongly scattered elastically at the interface with no memory of their original states as given by the DMM. For an epitaxial GaAs/AlGaAs sample at room temperature, the roughness usually extends to 1–3 atomic layers, or 3–9 Å, which is comparable to the dominant phonon wavelength^9,51^ and therefore, *α_ij_* is diffusive and the DMM can be applied. Under a high temperature condition, the DMM predicts that^9^

$$\alpha_{12}=\frac{c_{2}^{A}v_{2}^{A}}{c_{1}^{A}v_{1}^{A}+c_{2}^{A}v_{2}^{A}},$$ (15)

where the superscript *A* indicates that all parameters are evaluated based on a more realistic approximation of phonon dispersion relations,^9,51,52^ and $c_{1}^{A}$ ($c_{2}^{A}$) is much smaller than *c*_1_ (*c*_2_) used in Eq. (6) [Eq. (10)], because the optical phonon, which has a negligible contribution to the thermal transport, is excluded. Using this method, Chen^9,53^ has evaluated that $c_{1}^{A}=0.88\times 10^{6}$ J m^−3^ K and $v_{1}^{A}=1024$ m s^−1^. The dependency of *α*_12_ on the phonon frequency and incident angle is not considered in Eq. (15), which is typically assumed while using the DMM. Given *α*_12_ and $c_{1}^{A}v_{1}^{A}$, the TBR in the diffuse limit can then be calculated by^9,54^

$$R_{\mathrm{dif}}=\frac{4(1-0.5(\alpha_{12}+\alpha_{21}))}{\alpha_{12}c_{1}^{A}v_{1}^{A}}.$$ (16)

We use the same method developed for modeling the heat flow in GaAs/AlAs superlattices^51^ to evaluate the product of *c^A^* and *v^A^* ($\left\langle c^{A}v^{A}\right\rangle$) of all the acoustic phonon modes for GaAs and AlAs (the parameters are listed in Table I in Ref. 51). Due to the similarity of the phonon spectra of AlAs with those of GaAs,^58^ the above method should also be valid for Al_*x*_Ga_1−*x*_As. Likewise, the parameters for Al_*x*_Ga_1−*x*_As can be evaluated as

$$\begin{array}{c} {\left\langle c^{A}v^{A}\right\rangle }_{Al_{x}Ga_{1-x}As}=x\times {\left\langle c^{A}v^{A}\right\rangle }_{\mathrm{AlAs}}+(1-x)\times {\left\langle c^{A}v^{A}\right\rangle }_{\mathrm{GaAs}}. \end{array}$$ (17)

Using Eq. (15), *α*_12_ was evaluated to be 0.504 and has negligible temperature-dependency above 300 K. It is very close to the value of 0.5 for ideal DMM transport, because Al_0.25_Ga_0.75_As is almost identical to GaAs ($c_{1}^{A}v_{1}^{A}\approx c_{2}^{A}v_{2}^{A}$) in their mechanical properties. Finally, according to Eq. (16), *R_dif_* was evaluated to be $4.40\times 10^{-9}$ m^2^ K W^−1^ at 300 K, which is plotted as the dash-dotted line in Fig. 2(b). *R_dif_* only reduces about 2.5% as the lattice temperature increases from 300 K to 600 K, which justifies our treatment of *R_B_* as a temperature-independent parameter in the fitting. As shown in Fig. 2(b), the evaluated *R_dif_* matched two extracted *R_B_*'s from our experiment very well indicating that the heat transport mechanism in those two cases was diffusive. As a comparison, the *R_B_* for the smallest pumping fluence was much larger and we speculated that it reflected the effect of ballistic transport because the relevant phonon MFPs in GaAs and AlGaAs are estimated to be about 140 nm with a corresponding phonon relaxation time of about 140 ps around room temperature.^9^ Such extra TBR due to ballistic transport has been predicted by many theoretical studies^1,55^ and also been demonstrated in several experiments.^21,56,57^ When TBR due to the boundary condition^8,11,12^ is included, the heat equation based on Fourier's Law seems to give a correct prediction of the overall heat transport performance of the GaAs/AlGaAs heterojunction.

For the highest pumping fluence, the experimental temperature data decrease faster than the model fitting curve after 200 ps. Nevertheless, the extracted *R_B_* reduced further, which could not be explained. It may indicate the failure of Fourier's Law under such highly non-equilibrium and highly dynamical conditions, when the temperature change within the length scale of phonon MFP may become too large for the concept of local temperature and its gradient to be justified.^55^ Heat transport models based on the more fundamental Boltzmann equation may be needed in such conditions. Further investigations are needed to clarify our observation.

In conclusion, we investigated the thermal transport in a GaAs/AlGaAs heterostructure by directly monitoring the lattice temperature evolution in the time domain using ultrafast electron diffraction. The maximum lattice jump was 350 K above room temperature. Using heat equations based on Fourier's Law, *R_B_* was extracted and compared with previous theoretical studies. We found that *R_B_* decreased with increasing temperature imbalance across the interface. Additionally, we found that the heat transport model based on Fourier's Law with the modified boundary condition worked well in the diffusive transport region although the temperature changes in our experiment exceeded the small perturbation limit. When heating the sample up to 650 K, our model fitting started to deviate from the data at such a highly non-equilibrium temperature imbalance of 350 K across the interface.
